# The Patient Concerns Inventory—Head and Neck (PCI‐HN): Swedish Translation and Cultural Adaptation

**DOI:** 10.1111/scs.70326

**Published:** 2026-10-08

**Authors:** Ylva Tiblom Ehrsson, Sandra Einarsson, Göran Laurell, Simon N. Rogers, Lisa Tuomi, Caterina Finizia

**Affiliations:** ^1^ Department of Surgical Sciences, Section of Otorhinolaryngology and Head and Neck Surgery Uppsala University Uppsala Sweden; ^2^ Department of Food, Nutrition and Culinary Science Umeå University Umeå Sweden; ^3^ Wirral University Teaching Hospital Wirral UK; ^4^ Faculty of Health, Social Care and Medicine Edge Hill University Ormskirk UK; ^5^ Department of Health and Rehabilitation, Institute of Neuroscience and Physiology, Sahlgrenska Academy University of Gothenburg Gothenburg Sweden; ^6^ Department of Otorhinolaryngology, Head and Neck Surgery, Region Västra Götaland Sahlgrenska University Hospital Gothenburg Sweden; ^7^ Department of Otorhinolaryngology, Head and Neck Surgery, Institute of Clinical Sciences, Sahlgrenska Academy University of Gothenburg Gothenburg Sweden; ^8^ Department of Otorhinolaryngology, Region Västra Götaland Sahlgrenska University Hospital Gothenburg Sweden

**Keywords:** head and neck cancer, item prompt list, PCI‐HN

## Abstract

**Introduction:**

The Patient Concerns Inventory—Head and Neck (PCI‐HN) is an item prompt list used in clinical settings to help patients with head and neck cancer (HNC) and their families address diagnosis, treatment, recovery and well‐being issues. The PCI‐HN has two versions: PCI‐HN AD (after diagnosis) and PCI‐HN PT (post‐treatment). Today, no Swedish HNC‐specific prompt list exists.

**Aim:**

To translate and culturally adapt the two PCI‐HN into Swedish, ensuring conceptual, semantic and operational equivalence with the original English versions.

**Methods:**

The 10 steps suggested by the International Society for Health Economics and Outcomes Research (ISPOR) were followed. The two PCI‐HN were forward‐ and back‐translated; review by the research group reached consensus on translated versions. Patients and healthcare professionals evaluated the translated versions in three phases. After each phase, corrections were made and new versions were tested. Phase A involved a focus group interview with a national multi‐disciplinary team of healthcare professionals. Phase B included individual patient interviews. In phases A and B, the participants considered the understandability and relevance of each item in the PCI‐HN. In phase C, healthcare professionals rated perceived relevance on a questionnaire (content validity). After the research group had agreed on final versions, the PCI‐HN was reviewed by a proofreader.

**Results:**

Ten healthcare professionals (Phase A), 10 patients (Phase B) and 10 healthcare professionals (Phase C) participated. Adaptations were made to PCI‐HN AD and PCI‐HN PT to improve cultural, linguistic, and clarity aspects in response to the results. Content validity for the PCI‐HN was excellent.

**Conclusion:**

A translated and culturally adapted version of the PCI‐HN is now available in Swedish. Patients and healthcare professionals highlighted its usefulness. The PCI‐HN can now be implemented into the Swedish healthcare system to support patients and their families in raising issues related to the disease and treatment, and during the post‐treatment period.

## 1 Introduction

Head and neck cancer (HNC) is the sixth most common cancer type, both worldwide [1] and in Sweden, where approximately 1700 people are diagnosed annually [2]. Patients with HNC are a heterogeneous group having tumours of the pharynx, oral cavity, larynx, paranasal sinuses, nasal cavity or salivary glands [1]. The human papillomavirus (HPV) and a decrease in tobacco consumption have, in recent decades, partially revised the demographics of HNC to include not only older adults but also younger people of working age. The treatment of HNC depends on tumour site, tumour stage and comorbidities. Most patients receive radiotherapy, and in some cases with the addition of chemotherapy. Surgical treatment is commonly considered for several tumour sites. During surgical treatment, the primary tumour is removed and reconstruction can sometimes occur with, for example, an anterolateral thigh flap, radial free forearm flap, scapula or fibula free flap. In some cases, additional neck dissection of lymph nodes may be needed.

Acute radiation side effects are mainly related to damage to adjacent healthy tissues in the area of radiation treatment [3]. The addition of chemotherapy produces systemic toxicity and may exacerbate these local tissue reactions [4]. Despite advanced reconstructive surgery, extensive surgery often leads to functional difficulties due to impaired sensation, speech and chewing problems, dysphagia (swallowing problems), altered appearance and food residuals getting stuck in the oral cavity [5]. Symptoms of acute radiation side effects include taste and smell changes, mucositis (inflammation in the oral mucosa), xerostomia (dry mouth), dysphagia and pain in the mouth and throat [3, 4]. Xerostomia and the loss of taste may gradually subside after treatment, but for many patients these become lifelong irreversible problems [3]. Late HNC radiation side effects include serious gum inflammation (periodontitis), caries, trismus (painful restriction in opening the mouth), dysphagia and osteoradionecrosis (breakdown of the jawbone) [6, 7, 8], which often have a negative secondary impact on quality of life [9]. Chemotherapy can also cause central nervous system or peripheral nervous system toxicity and hearing loss, which can be irreversible [10, 11]. Patients may also suffer from fatigue, depression and anxiety [8]. In addition, patients may have nutritional problems that affect them not only physically, but also socially due to their avoidance of socializing and eating with others [12]. Consequently, understanding the diverse experiences of patients with HNC throughout the treatment journey—from pre‐treatment to post‐treatment phases—is paramount. By exploring patients' reported challenges and problems, both immediate and long‐term, we can gain a comprehensive understanding of their unique struggles, concerns and unmet needs. Multidisciplinary support is crucial in addressing their multifaceted issues, and the use of a patient‐reported outcome (PRO) measure to help identify patients who need additional help would be extremely helpful. Currently, there is no HNC‐specific prompt list available in Swedish.

The Patient Concerns Inventory—Head and Neck (PCI‐HN) was developed in the UK in the early 2000s [13]. It is an item prompt list (a structured checklist of predefined concerns) designed to be used in clinical settings to help patients and their families address issues about the diagnosis, treatment, recovery and their well‐being that they would like to discuss with physicians, nurses or other healthcare professionals [14]. The PCI‐HN has received interest and gained acceptance in many countries around the world and its use is feasible in clinical care [15, 16] but, most importantly, it has shown the potential to improve patients' health‐related quality of life (HRQoL) [17, 18]. The aim of the present study was to conduct Swedish translation and cultural adaptation of the two versions of the Patient Concerns Inventory—Head and Neck (PCI‐HN) item prompt list by following a structured process to ensure conceptual, semantic and operational equivalence to the original English versions.

## 2 Material and Methods

### 2.1 The Patient Concerns Inventory—Head and Neck

There are two versions of the PCI‐HN; one for patients to fill in after diagnosis, at the time of treatment decision‐making and before treatment (AD), and another for patients to fill in during and after treatment (PT). The PCI‐HN AD has 75 items grouped into seven domains: cause of cancer; treatment‐related; what will I be like; follow‐up; social care and social well‐being; psychological, emotional and spiritual well‐being; physical and functional well‐being; and other (free text). At the end of the PCI‐HN AD is a list of 18 healthcare professionals that might be able to help the patient. The PCI‐HN PT has 56 items grouped into five domains: physical and functional well‐being; treatment‐related; social care and social well‐being; psychological, emotional and spiritual well‐being; and other (free text). At the end of the PCI‐HN PT is a list of 18 healthcare professionals who the patient can indicate that they wish to talk to during treatment or their post‐treatment recovery. The list of healthcare professionals in both versions of the PCI‐HN can be changed depending on what is available at individual centres and is also dependent on shared roles within the multidisciplinary team.

### 2.2 Translation and Cultural Adaptation

The translation and cultural adaptation of PCI‐HN AD and PCI‐HN PT into Swedish was carried out by a multidisciplinary research group (physicians, nurse, speech and language therapist, dietitian) with long‐standing expertise in HNC, from clinical practice and/or research. It followed the 10 steps described by the guideline ‘Principles of Good Practice for the Translation and Cultural Adaptation Process for Patient‐Reported Outcomes (PRO) Measures’ [19] from the Professional Society for Health Economics and Outcomes Research, ISPOR.

#### 2.2.1 Steps 1–6

Step 1 (preparation) was met by the composition of the research group, which included the person (S.R.) who developed the PCI‐HN in the UK. The research group was a multidisciplinary group including one HNC nurse, one dietitian, one speech and language therapist, and three ear, nose and throat (ENT) physicians. In Steps 2–6, the translation and cultural adaptation of PCI‐HN AD and PCI‐HN PT followed a comprehensive process of forward translation, reconciliation, back translation and review and harmonization. In Steps 2 and 3, the PCI‐HN AD and PCI‐HN PT were translated from English to Swedish by two researchers (Y.T.E. and S.E.) and then reviewed by the research group to reach a consensus. Step 4 involved translation of the Swedish PCI‐HN back to English by a translator. In Step 5, the translation was reviewed and discussed by the research group. In Step 6, all translations were compared with the original versions by the research group to reach consensus on final versions of the PCI‐HN to be tested in Step 7. This consensus was achieved through interactive group discussions, which facilitated the resolution of discrepancies and the refinement of wording.

#### 2.2.2 Step 7

In Step 7, the translated PCI‐HN was tested by patients and healthcare professionals in a cross‐sectional study in 2024. This was performed by the first author (Y.T.E.) in three phases: (A) Focus group interview with healthcare professionals (‘expert panel’); (B) Individual interviews with patients; and (C) Testing the content validity of the PCI‐HN with help from healthcare professionals. Participants were recruited using a convenience sampling procedure. The PCI‐HN was adjusted after each phase of Step 7 and the new versions of the PCI‐HN were tested in the next phase. The adjustments were made by two researchers (Y.T.E. and S.E.) followed by a review by the research group. No items were added or removed to secure alignment with the original English versions; this enables international research collaborations and comparisons using the PCI‐HN. However, the list of healthcare professionals available for patients to see can vary depending on the setting or country and may be changed accordingly. Therefore, healthcare professionals were added to or removed from the list of healthcare professionals at the end of the PCI‐HN to culturally adapt the Swedish versions of the PCI‐HN. Hence, this part does not align exactly with the original English version of the PCI‐HN.

##### 2.2.2.1 Ethical Approval and Informed Consent

The study was approved by the Swedish Ethical Review Authority (Dnr 2024–00281‐01) and all participants gave signed informed consent before participation.

##### 2.2.2.2 Participants and Data Collection

In phase A, a national multidisciplinary group of healthcare professionals (‘expert panel’) were recruited to a focus group interview led by the first author (Y.T.E.) [20]. Before the interview, the participants were asked to review the PCI‐HN AD and PCI‐HN PT closely. During the interview, the moderator asked questions about the understandability and relevance of each item in the PCI‐HN. If comprehensibility was poor, the participants were asked to explain why, for example if this was due to inaccurate translation or a concept being not culturally relevant. The focus group interview was recorded and additional written notes were made by the moderator. A short questionnaire was used to gather information about participants' characteristics (profession, years within the profession and years working with HNC patients).

In phase B, patients were recruited from one county hospital in the southern part of Sweden. Inclusion criteria were Swedish‐speaking patients, > 18 years old with HNC or being treated for HNC. The exclusion criteria were severe cognitive impairment, inability to understand the Swedish language or other factors causing inability to participate in studies, for example, severe comorbidities and logistical barriers. Patients were asked to participate in the study by healthcare professionals in contacts during regular care. Individual interviews were led by the first author (Y.T.E). Before the interview, the patients were asked to review the PCI‐HN AD and PCI‐HN PT closely. During the interview, the researcher asked questions about the understandability and relevance of each item of the PCI‐HN. If comprehensibility was poor, the patients were asked to explain why and, if possible, give suggestions for improvement. The interviews were recorded, and additional written notes were made by the researcher. A short additional questionnaire was used to gather information about the patient's characteristics (sex, age, education level and current occupation); information regarding diagnosis and treatment was obtained from the medical records.

In phase C, healthcare professionals assessed the perceived relevance (content validity) of the PCI‐HN AD and PCI‐HN PT, respectively [21, 22]. Healthcare professionals were approached and those who agreed to participate were instructed to complete a questionnaire to rate the perceived relevance of each item in the PCI‐HN AD and PCI‐HN PT, respectively, using a four‐point Likert scale (1 = very irrelevant, 2 = irrelevant, 3 = relevant and 4 = very relevant). Open‐ended questions allowed the healthcare professionals to freely state concerns related to the PCI‐HN. Additional questions were used to gather information about participants' characteristics (profession, years within the profession and years working with HNC patients).

##### 2.2.2.3 Analyses

The qualitative data from the focus group interview (Phase A) and individual interviews (Phase B) were analysed by underlining key statements made by the participants regarding suggested changes to the PCI‐HN. Quantitative data from the questionnaire (Phase C) were used to assess content validity. Content Validity Index (CVI) was calculated for each item (I‐CVI) of the PCI‐HN [21, 22] by dividing the number of participants giving Likert scale ratings 3 and 4 by the total number of participants. Scale‐CVI (S‐CVI) was calculated using the average of the I‐CVIs. Cut‐off standards for I‐CVI and S‐CVI, respectively, were used as a reference [22]. I‐CVIs > 0.78 are defined as excellent, whereas I‐CVIs < 0.78 need further analysis. S‐CVIs ≥ 0.90 are considered excellent and S‐CVIs between 0.80–0.89 are considered acceptable. Participant characteristics were presented using descriptive statistics: frequency (percent) and mean (min, max). Quantitative data were analysed using Microsoft Excel.

## 3 Results

### 3.1 Phase A: Focus Group Interview With Healthcare Professionals

The participants (‘expert panel’) in the focus group interview were: an ear‐nose‐throat (ENT) physician, a specialist nurse, a dietitian, a speech and language therapist and a rehabilitation physician. The mean number of years in their profession was 20 years (min 9 years, max 29 years) and mean number of years working with HNC patients was 13 years (min 4 years, max 22 years).

The focus group interview resulted in minor cultural adaptations (*n* = 4 and *n* = 2), minor linguistic (*n* = 4 and *n* = 0) and minor clarification changes (*n* = 16 and *n* = 17) to PCI‐HN AD and PCI‐HN PT, respectively. For example, for both versions, the explanatory text was made clearer after suggestions from the ‘expert panel’. ‘Research nurse’ was added to both versions as a profession that patients could meet after diagnosis as well as after treatment, and ‘Sexologist/fertility expert’ was added to PCI‐HN AD. Some healthcare professionals in the ‘expert panel’ raised concerns about the last part of PCI‐HN PT where patients can highlight professions that they would like to talk to. The text in relation to this was clarified so that patients would understand the list of professions to be a ‘wish‐list’ rather than an ‘on demand‐list’. Many of the participants in the focus group interview highlighted the benefits of using an PCI‐HN that specifically identifies what is important to the patient and what they would like to talk about.

### 3.2 Phase B: Individual Interviews With Patients

The mean age of the patients participating in individual interviews (five females and five males) was 64 years (min 50 years, max 77 years). Their education level was elementary school (*n* = 7) or university (*n* = 3). Three patients were currently working and seven were retired. Seven patients had a tumour of the oropharynx and three a tumour of the oral cavity. According to the Union for International Cancer Control's (UICC) 8, the patients with tumours of the oropharynx had Stage I (*n* = 5), Stage II (*n* = 1) or Stage III (*n* = 1) tumours. The patients with tumours of the oral cavity had Stage III (*n* = 1) or Stage IV (*n* = 2) tumours. Treatment modality was radiotherapy (*n* = 5), surgery with postoperative radiotherapy (*n* = 1), or chemoradiotherapy (*n* = 2). In addition, at the time of the interview, one patient had received surgery for a primary tumour and then surgery with postoperative radiotherapy for residual disease, and one patient had received surgery and was planned for additional radiotherapy. The mean time from the end of treatment to follow‐up was 20 months (min 1 month, max 55 months).

The interviews with patients resulted in minor cultural adaptations (*n* = 1 and *n* = 2) and minor clarification changes (*n* = 4 and *n* = 4) to PCI‐HN AD and PCI‐HN PT, respectively. For example, the abbreviation ‘HPV’ was unfamiliar to some participants. Therefore, we also chose to write out the full form of the abbreviation. The term ‘neck dissection’ was replaced with simpler language, ‘type of surgery’, due to confusion among patients. Although ‘feeding tube’ was not a previously known word to two patients, the original terminology was maintained. Some participants noted that certain emotional descriptors, such as ‘Angry’, did not resonate with their experiences; however, these were kept for consistency with the original questionnaire. Also, ‘Activity’ was revised to ‘Physical activity’ to avoid misinterpretation, as some associated the term with cultural rather than physical engagement. Additionally, a word (oedema and hearing, respectively) was added in brackets to both versions for two professions (lymphatic therapist and audiologist, respectively) to make the function of these professions clearer to the patient.

Regarding the last part of the PCI‐HN PT, where patients can highlight professions that they would like to meet, the patients understood this as being a ‘wish list’ of professions to talk to if possible, not something that they could demand. All patients stated that they would like to use the PCI‐HN. Other more general comments made by the patients were that the PCI‐HN was ‘quick and easy to fill in’, that it ‘addressed problems important for the patient’, and that it could work as a ‘reminder checklist’ for the patient to complete at home or while sitting in the waiting room.

### 3.3 Phase C: Questionnaire to Healthcare Professionals

The professions of the participants answering the questionnaire were: physicians (*n* = 4), nurses (*n* = 4), a dietitian and a speech and language therapist. The mean number of years in the profession was 26 years (min 14 years, max 40 years) and mean number of years working with patients with HNC was 17 years (min 6 years, max 40 years). In addition, one physician gave verbal comments about the understandability and relevance of each item after closely reviewing the PCI‐HN AD and PCI‐HN PT.

When assessing perceived relevance (content validity), most items in the PCI‐HN AD and PCI‐HN PT received excellent content validity ratings (I‐CVI > 0.78) (Tables 1 and 2) [22]. Seven items in PCI‐HN AD (Spirituality/religious aspects, Personality and temperament, Bowel habit, Regurgitation, Lymphatic therapist (oedema) and Acupuncturist) and one item in PCI‐HN PT (Spirituality/religious aspects) had an I‐CVI < 0.78, indicating low content validity. The S‐CVI was 0.91 and 0.96 for the PCI‐HN AD and PCI‐HN PT, respectively, indicating excellent content validity.

**TABLE 1:** Item and Scale Content Validity Indexes (I‐CVI, S‐CVI) for the Patient Concerns Inventory—Head and Neck (PCI‐HN) after diagnosis (AD).

Item PCI‐HN AD	Expert 1	Expert 2	Expert 3	Expert 4	Expert 5	Expert 6	Expert 7	Expert 8	Expert 9	Expert 10	Experts with √	I‐CVI^a^
*Cause of cancer*												
Lifestyle issues (smoking/alcohol)	√	√	√	√	√	√	√	√	√	√	10	1.00
HPV	√	√	√	√	√		√	√	√	√	9	0.90
Other	√	√		√	√	√	√	√	√	√	9	0.90
*Treatment related*												
Investigations needed (MRI/CT)	√	√	√	√	√	√	√	√	√	√	10	1.00
Investigation results	√	√	√	√	√	√	√	√	√	√	10	1.00
Cancer treatment: what choices of treatment are available	√	√	√	√	√	√	√	√	√	√	10	1.00
Treatment intent: cure or palliation	√	√	√	√	√	√	√	√	√	√	10	1.00
Clinical trials—contributing to research	√	√		√	√		√	√	√	√	8	0.80
Nutrition/feeding tube/PEG	√	√		√	√	√	√	√	√	√	9	0.90
What are the side effects/toxicity/complications of treatments	√	√	√	√	√	√	√	√	√	√	10	1.00
Surgery: length of stay in hospital, neck dissection, laser, free flap	√	√	√	√	√	√	√	√	√	√	10	1.00
Radiotherapy: mould room, number of treatments, sore mouth and skin	√	√	√	√	√	√	√	√	√	√	10	1.00
Chemotherapy: number of treatments, deafness, nausea	√	√	√	√	√	√	√	√	√	√	10	1.00
What is the chance of cure	√	√	√	√	√	√	√	√	√	√	10	1.00
When will I know I am all clear—when will I know treatment has been successful	√	√	√	√	√	√	√	√	√	√	10	1.00
What treatments are available if the cancer comes back	√	√		√	√		√	√	√	√	8	0.80
How long am I likely to live	√	√	√	√	√		√	√	√	√	9	0.90
Waiting time for commencement of treatment	√	√	√	√	√	√	√	√	√	√	10	1.00
Duration of treatment	√	√	√	√	√	√	√	√	√	√	10	1.00
Dental check up	√	√		√	√		√	√	√	√	8	0.80
*What will it be like*												
What are the main issues that patients find important, e.g., speech, swallowing	√	√	√	√	√		√	√	√	√	9	0.90
What sort of ‘quality of life’ do patients report	√	√	√	√	√		√	√	√	√	9	0.90
*Follow‐up*												
What sort of follow up do I need/how often/scans/tests	√	√	√	√	√	√	√	√	√	√	10	1.00
Patient and Carer Support Group/meet other patients	√	√	√	√	√		√	√	√	√	9	0.90
*Social care and social well‐being*												
Carer	√	√	√	√	√	√	√	√	√	√	10	1.00
Dependants/children	√	√	√	√	√	√	√	√	√	√	10	1.00
Finance/money	√	√	√	√	√	√	√	√	√	√	10	1.00
Benefits/what's free and what's not	√	√	√	√	√	√	√	√	√	√	10	1.00
Time off work	√	√	√	√	√	√	√	√	√	√	10	1.00
Home care/District nurse	√	√		√	√	√	√	√	√	√	9	0.90
Recreation	√	√		√	√		√	√	√	√	8	0.80
Relationships	√	√	√	√	√		√	√	√	√	9	0.90
Speech/voice/being understood	√	√	√	√	√	√	√	√	√	√	10	1.00
Support for my family	√	√	√	√	√		√	√	√	√	9	0.90
*Psychological, emotional and spiritual well‐being*												
Appearance	√	√	√	√	√	√	√	√	√	√	10	1.00
Angry	√	√		√	√		√	√	√	√	8	0.80
Anxiety	√	√	√	√	√	√	√	√	√	√	10	1.00
Coping	√	√	√	√	√		√	√	√		8	0.80
Depression	√	√	√	√	√	√	√	√	√	√	10	1.00
Fear of adverse events	√	√		√	√	√	√	√	√	√	9	0.90
Intimacy	√	√	√	√	√	√	√	√	√	√	10	1.00
Memory	√	√		√	√	√	√	√	√		8	0.80
Mood	√	√	√	√	√	√	√	√	√		9	0.90
Self‐esteem	√	√		√	√		√	√	√	√	8	0.80
Sexuality	√	√		√	√	√	√	√	√	√	9	0.90
Spirituality/religious aspects	√			√	√		√				4	0.40
Personality and temperament	√	√		√	√		√	√	√		7	0.70
*Physical and functional well‐being*												
Activity	√	√	√	√	√	√	√	√	√	√	10	1.00
Appetite	√	√	√	√	√	√	√	√	√	√	10	1.00
Bowel habit	√			√	√		√	√	√		6	0.60
Chewing/eating	√	√	√	√	√	√	√	√	√	√	10	1.00
Coughing	√	√		√	√		√	√	√		7	0.70
Dental; health/teeth	√	√		√	√	√	√	√	√	√	9	0.90
Dry mouth	√	√	√	√	√	√	√	√	√	√	10	1.00
Energy levels	√	√	√	√	√	√	√	√	√	√	10	1.00
Fatigue/tiredness	√	√	√	√	√	√	√	√	√	√	10	1.00
Hearing	√	√		√	√	√	√	√	√		8	0.80
Indigestion	√	√		√	√	√	√	√	√		8	0.80
Mobility	√	√		√	√	√	√	√	√	√	9	0.90
Mouth opening	√	√	√	√	√	√	√	√	√	√	10	1.00
Mucus	√	√		√	√	√	√	√	√		8	0.80
Nausea	√	√	√	√	√	√	√	√	√		9	0.90
Pain in the head and neck	√	√	√	√	√	√	√	√	√	√	10	1.00
Pain elsewhere	√	√		√	√		√	√	√	√	8	0.80
Regurgitation	√	√		√	√		√	√	√		7	0.70
Salivation	√	√	√	√	√	√	√	√	√	√	10	1.00
Shoulder	√	√		√	√		√	√	√	√	8	0.80
Sleeping	√	√	√	√	√	√	√	√	√	√	10	1.00
Smell	√	√	√	√	√	√	√	√	√	√	10	1.00
Sore mouth	√	√	√	√	√	√	√	√	√	√	10	1.00
Swallowing	√	√	√	√	√	√	√	√	√	√	10	1.00
Swelling	√	√	√	√	√	√	√	√	√	√	10	1.00
Taste	√	√	√	√	√	√	√	√	√	√	10	1.00
Vomiting/sickness	√	√	√	√	√	√	√	√	√		9	0.90
Weight	√	√	√	√	√		√	√	√	√	9	0.90
*Professions*												
Dental hygienist	√	√	√	√	√		√	√	√	√	9	0.90
Dentist	√	√	√	√	√	√	√	√	√	√	10	1.00
Dietitian	√	√	√	√	√	√	√	√	√	√	10	1.00
Speech (swallow) and language therapist	√	√	√	√	√	√	√	√	√	√	10	1.00
Occupational therapist	√	√		√	√		√	√	√	√	8	0.80
Physiotherapist	√	√	√	√	√	√	√	√	√	√	10	1.00
Lymphatic therapist (oedema)^b^	√	√		√			√	√	√	√	7	0.70
Acupuncturist^b^	√	√		√			√	√	√		6	0.60
Audiologist	√	√	√	√	√	√	√	√	√	√	10	1.00
Sexologist/fertility expert^b^	√	√		√	√		√	√	√	√	8	0.80
Oncologist/Radiotherapist	√	√	√	√	√	√	√	√	√	√	10	1.00
Clinical Nurse Specialist	√	√	√	√	√	√	√	√	√	√	10	1.00
Research nurse^b^	√	√		√	√		√	√	√	√	8	0.80
Surgeon	√	√	√	√	√	√	√	√	√	√	10	1.00
Social worker	√	√		√	√	√	√	√	√	√	9	0.90
Financial advisor	√	√	√	√	√		√	√	√	√	9	0.90
Nursing staff	√	√		√	√	√	√	√	√	√	9	0.90
General practitioner	√	√		√	√	√	√	√	√	√	9	0.90
Home care^b^	√	√	√	√	√	√	√	√	√	√	10	1.00
Chaplain	√	√		√	√		√	√	√	√	8	0.80
Clinical psychologist	√	√	√	√	√		√	√	√	√	9	0.90
Emotional support therapist	√	√	√	√	√	√	√	√	√	√	10	1.00
											S‐CVI^c^	0.91

*Note:* Swedish translation and cultural adaptation as perceived by 10 healthcare professionals. The healthcare professionals rated each item of the instrument on a four‐point Likert scale (√ shows number that rated 3 = relevant or 4 = very relevant).

^a^ Item Content validity Index (I‐CVI): number of healthcare professionals giving Likert scale ratings 3 and 4 divided by the total number of healthcare professionals. The I‐CVI cut‐off for excellent is ≥ 0.78.

^b^ Item added in the Swedish version.

^c^ Scale Content Validity Index (S‐CVI): Average of I‐CVIs. The S‐CVI cut‐off for acceptability is ≥ 0.80–0.89 and is considered excellent at ≥ 0.90.

**TABLE 2:** Item and Scale Content Validity Index (I‐CVI, S‐CVI) for the Patient Concerns Inventory—Head and Neck (PCI‐HN) post treatment (PT).

Item PCI‐HN PT	Expert 1	Expert 2	Expert 3	Expert 4	Expert 5	Expert 6	Expert 7	Expert 8	Expert 9	Expert 10	Experts with √	I‐CVI^a^
*Physical and functional well‐being*												
Activity	√	√	√	√	√	√	√	√	√	√	10	1.00
Appetite	√	√	√	√	√	√	√	√	√	√	10	1.00
Bowel habit	√	√	√	√	√		√	√	√	√	9	0.90
Breathing	√	√	√	√	√	√	√	√	√	√	10	1.00
Chewing/eating	√	√	√	√	√	√	√	√	√	√	10	1.00
Coughing	√	√	√	√	√		√	√	√	√	9	0.90
Dental; health/teeth	√	√	√	√	√	√	√	√	√	√	10	1.00
Dry mouth	√	√	√	√	√	√	√	√	√	√	10	1.00
Energy levels	√	√	√	√	√		√	√	√	√	9	0.90
Fatigue/tiredness	√	√	√	√	√	√	√	√	√	√	10	1.00
Hearing	√	√	√	√	√	√	√	√	√	√	10	1.00
Indigestion	√	√	√	√	√	√	√	√	√	√	10	1.00
Mobility	√	√	√	√	√	√	√	√	√	√	10	1.00
Mouth opening	√	√	√	√	√	√	√	√	√	√	10	1.00
Mucus	√	√	√	√	√	√	√	√	√	√	10	1.00
Nausea	√	√	√	√	√	√	√	√	√	√	10	1.00
Pain in the head and neck	√	√	√	√	√	√	√	√	√	√	10	1.00
Pain elsewhere	√	√	√	√	√	√	√	√	√	√	10	1.00
Regurgitation	√	√	√	√	√		√	√	√	√	9	0.90
Salivation	√	√	√	√	√	√	√	√	√	√	10	1.00
Shoulder	√	√	√	√	√		√	√	√	√	9	0.90
Sleeping	√	√	√	√	√	√	√	√	√	√	10	1.00
Smell	√	√	√	√	√	√	√	√	√		9	0.90
Sore mouth	√	√	√	√	√	√	√	√	√	√	10	1.00
Swallowing	√	√	√	√	√	√	√	√	√	√	10	1.00
Swelling	√	√	√	√	√	√	√	√	√	√	10	1.00
Taste	√	√	√	√	√	√	√	√	√	√	10	1.00
Vomiting/sickness	√	√	√	√	√	√	√	√	√	√	10	1.00
Weight	√	√	√	√	√	√	√	√	√	√	10	1.00
*Treatment related*												
Cancer treatment	√	√	√	√	√	√	√	√	√	√	10	1.00
Regret about treatment	√	√	√	√	√		√	√	√		8	0.80
PEG tube	√	√	√	√	√	√	√	√	√	√	10	1.00
Wound healing	√	√	√	√	√	√	√	√	√	√	10	1.00
*Social care and social well‐being*												
Carer	√	√	√	√	√	√	√	√	√	√	10	1.00
Dependents/children	√	√	√	√	√	√	√	√	√	√	10	1.00
Financial benefits	√	√	√	√	√		√	√	√	√	9	0.90
Home care/District nurse	√	√	√	√	√	√	√	√	√	√	10	1.00
Lifestyle issues (smoking/alcohol)	√	√	√	√	√	√	√	√	√	√	10	1.00
Recreation	√	√	√	√	√		√	√	√	√	9	0.90
Relationships	√	√	√	√	√	√	√	√	√	√	10	1.00
Speech/voice/being	√	√	√	√	√	√	√	√	√	√	10	1.00
Understood	√	√	√	√	√	√	√	√	√	√	10	1.00
*Psychological, emotional and spiritual well‐being*												
Appearance	√	√	√	√	√	√	√	√	√	√	10	1.00
Angry	√	√	√	√	√		√	√	√	√	9	0.90
Anxiety	√	√	√	√	√	√	√	√	√	√	10	1.00
Coping	√	√	√	√	√		√	√	√	√	9	0.90
Depression	√	√	√	√	√	√	√	√	√	√	10	1.00
Fear of the cancer coming back	√	√	√	√	√	√	√	√	√	√	10	1.00
Fear of adverse events	√	√	√	√	√	√	√	√	√	√	10	1.00
Intimacy	√	√	√	√	√	√	√	√	√	√	10	1.00
Memory	√	√		√	√	√	√	√	√	√	9	0.90
Mood	√	√	√	√	√		√	√	√	√	9	0.90
Self‐esteem	√	√	√	√	√	√	√	√	√	√	10	1.00
Sexuality	√	√	√	√	√	√	√	√	√	√	10	1.00
Spiritual/religious aspects	√	√		√	√		√	√			6	0.60
Personality and temperament	√	√	√	√	√		√	√	√		8	0.80
*Professions*												
Dental hygienist	√	√	√	√	√		√	√	√	√	9	0.90
Dentist	√	√	√	√	√	√	√	√	√	√	10	1.00
Dietitian	√	√	√	√	√	√	√	√	√	√	10	1.00
Speech (swallow) and language therapist	√	√	√	√	√	√	√	√	√	√	10	1.00
Occupational therapist	√	√	√	√	√		√	√	√	√	9	0.90
Physiotherapist	√	√	√	√	√	√	√	√	√	√	10	1.00
Lymphatic therapist (oedema)^b^	√	√	√	√	√		√	√	√	√	9	0.90
Acupuncturist^b^	√	√	√	√	√		√	√	√		8	0.80
Audiologist	√	√	√	√	√	√	√	√	√	√	10	1.00
Sexologist^b^	√	√	√	√	√		√	√	√	√	9	0.90
Oncologist/Radiotherapist	√	√	√	√	√	√	√	√	√	√	10	1.00
Clinical Nurse Specialist	√	√	√	√	√	√	√	√	√	√	10	1.00
Research nurse^b^	√	√		√	√		√	√	√	√	8	0.80
Surgeon	√	√	√	√	√	√	√	√	√	√	10	1.00
Social worker	√	√	√	√	√		√	√	√	√	9	0.90
Financial advisor	√	√	√	√	√		√	√	√	√	9	0.90
Nursing staff	√	√	√	√	√		√	√	√	√	9	0.90
General practitioner	√	√	√	√	√		√	√	√	√	9	0.90
Home care^b^	√	√	√	√	√	√	√	√	√	√	10	1.00
Chaplain	√	√	√	√	√		√	√	√	√	9	0.90
Clinical psychologist	√	√	√	√	√		√	√	√	√	9	0.90
Emotional support therapist	√	√	√	√	√	√	√	√	√	√	10	1.00
											S‐CVI^c^	0.96

*Note:* Swedish translation and cultural adaptation as perceived by 10 healthcare professionals. The healthcare professionals rated each item of the instrument on a four‐point Likert scale (√ shows number that rated 3 = relevant or 4 = very relevant).

^a^ Item Content validity Index (I‐CVI): number of healthcare professionals giving Likert scale ratings 3 and 4 divided by the total number of healthcare professionals. The I‐CVI cut‐off for excellent is ≥ 0.78.

^b^ Item added in the Swedish version.

^c^ Scale Content Validity Index (S‐CVI): Average of I‐CVIs. The S‐CVI cut‐off for acceptability is ≥ 0.80–0.89 and is considered excellent at ≥ 0.90.

The verbal comments and additional written comments from the open‐ended questions on the questionnaire resulted in minor cultural adaptations (*n* = 5 and *n* = 1), minor linguistic changes (*n* = 3 and *n* = 2) and minor clarification changes (*n* = 14 and *n* = 6) to PCI‐HN AD and PCI‐HN PT, respectively. ‘Phoniatrician’ was added to the list of healthcare professionals at the end of both versions.

### 3.4 Steps 8–10

After the cognitive debriefing in Step 7, the research group agreed on a final version (Step 8). During this process, the research group agreed on keeping items with low content validity (I‐CVI < 0.78) to ensure consistency with the original English version of the PCI‐HN. The Swedish PCI‐HN was thereafter reviewed by a proofreader (Step 9) to correct any grammatical errors. Step 10 is finalized through the scientific publication of the translation and cultural adaptation process.

## 4 Discussion

Strategies to enhance communication with, and the involvement of, patients with HNC and their caregivers in treatment and care are much needed. The original English version of the PCI‐HN has been shown to improve HRQoL in several studies [17, 18, 23]. In this study, the PCI‐HN AD and PCI‐HN PT were successfully translated and culturally adapted into Swedish by following the systematic methodology described by ISPOR [19]. The availability of the PCI‐HN adapted to a Swedish setting is an important step towards implementing an item prompt list in clinical practice that is specifically designed for patients with HNC. The problems that patients with HNC experience—both during treatment and long‐term post‐treatment—are multifaceted in nature, so using a patient reported outcome (PRO) measure that focuses on each patient's specific needs in a simple and effective way is crucial. Patients interviewed in the study confirmed that they would like to use the PCI‐HN, and the healthcare professionals perceived the PCI‐HN as relevant to their clinical practice, as shown by the excellent ratings for content validity. This is also confirmed by Shunmugasundaram et al., who, due to its excellent content validity, recommend the PCI‐HN for assessing unmet needs in patients with HNC [16].

In Sweden, patients treated for HNC are followed in a structured manner through physician appointments for up to 5 years post‐treatment according to national guidelines. To make these follow‐ups more efficient, and to limit the use of healthcare resources, item prompt lists can be helpful during the consultation to guide patients and help them address issues that are important to them. There is currently no holistic Swedish PRO measure that can be used in the clinical setting which captures all essential dimensions important for patients with HNC over time that is, disease, treatment and recovery, as well as physical, psychological, social and spiritual well‐being. The PRO measures that are available often measure specific dimensions/areas, such as the Head and Neck Patient Symptom Checklist (HNSC) [24, 25] that specifically address nutrition impact symptoms, the Gothenburg Trismus Questionnaire (GTQ) [26, 27] that focuses on trismus or the M.D. Anderson Dysphagia Inventory (MDADI) that focuses on dysphagia [28, 29]. There are also PRO measures that, due to their design, are more suitable for research purposes than clinical practice, for example the European Organization for Research and Treatment of Cancer Quality of Life Questionnaire‐Core 30 (EORTC QLQ‐C30) [30]. The Swedish version of the PCI‐HN will, therefore, make an important contribution to the clinical setting in Sweden. Nevertheless, it is important to note that the validated Swedish translation of the PCI‐HN is well‐suited to research purposes, to use as an outcome measure, and for comparing results across different countries regarding diagnosis, treatment, recovery and well‐being issues addressed by patients [18].

Both versions of the PCI‐HN include items specifically designed for patients with HNC. The use of an item prompt list in clinical practice has been shown to improve patients' HRQoL [17, 18] and increase the number of rehabilitation referrals [31], as well as promote holistic multidisciplinary support [32]. The PCI‐HN has also been shown to be a low‐cost intervention compared to a non‐PCI treatment pathway, and clinicians found it easy to incorporate into their consultations after minimal training [33]. In addition, the items of the PCI‐HN have been shown to be suitable for long‐term follow‐up consultations [34] and can, therefore, help healthcare professionals to understand and highlight patients' needs for extra support in the post‐treatment period and long‐term. Previous studies have, for example, shown that patients feeling distressed [35] or patients with lower scorings on HRQoL questionnaires [36] often report issues with a higher number of items on the PCI‐HN. An item prompt list, such as the PCI‐HN, is also a suitable tool for use by all members of the head and neck team; it can guide healthcare professionals in patient communication dynamics and in how interventions should be designed to properly support patients suffering from long‐term sequelae. If used correctly, the PCI‐HN does not necessarily lead to longer consultations [17, 37].

Patients with cancer express unmet information needs [38], and often seek more information than is regularly provided by healthcare professionals [39]. A study of 188 patients with HNC showed that patients wanted to receive more information, especially about future conditions (64.3%), treatments (50%) and the diagnosis (45%) [40]. In addition, 50% of the patients wanted to connect with others who had similar experiences. These results emphasize the need for better communication and psychosocial support in patient care. Adequate information is important for the patient to be able to gain control, reduce anxiety, improve compliance, create realistic expectations, promote self‐care and generate feelings of safety and security [41]. Shared decision‐making is considered a cornerstone of modern healthcare [42], and patients who are not sufficiently informed will not be able to become actively involved in their own care or decisions. A study by Gattellari et al. [43] on 233 patients with cancer showed that those who perceived having a shared role in decision‐making were more satisfied with the consultation, the information given about treatment and the emotional support they had received. Patients who ask questions generally receive more information [44], so one possible strategy to increase the amount of information given is to encourage the patient to ask more questions. The PCI‐HN has the potential to facilitate information transfer and therefore improve the shared decision‐making process to better align treatment decisions with patients' values and preferences.

Given the many advantages shown for using PCI‐HN, successful implementation into the Swedish healthcare system would likely create positive effects for both patients and healthcare professionals. Nationwide use of the PCI‐HN would facilitate national analysis of data on HNC patients' expressed needs during the trajectory of care. The healthcare system in Sweden is decentralized to 21 regions and for certain specialised treatments, such as those for specific types of cancer including HNC, care is mainly centralised to the university hospitals. To be able to use the PCI‐HN to its full extent, it is important to properly engage the healthcare professionals who will use the PCI‐HN in their daily practice. The relative simplicity of the PCI means that it is also straightforward to adapt to digital use and remote access.

The methodology used in the present study followed the steps suggested in ISPOR for how PRO measures should be translated and culturally adapted [19], which is a major strength. The composition of the research group is another strength, since its multidisciplinary nature secured rigour in the decisions taken during each step of ISPOR. Another strength is the inclusion of patients in the cognitive debriefing in Step 7, ensuring that all items of importance for patients in a Swedish context are addressed and properly explained in the Swedish PCI‐HN. We included a convenience sample of 10 participants per phase, which always comes with a risk of selection bias, but by including different professionals and patients with different diagnoses, sex, etc., we wanted to capture different perspectives and experiences. The cultural, linguistic and clarity adaptations made to the translated PCI‐HN in the different phases were only minor and did not alter the core concept, which means that the Swedish versions are comparable to the English versions. Available healthcare professionals may vary between different hospitals and also, as in this case, between countries. The Swedish list of healthcare professionals is notably more extensive and system‐oriented than the English version (listing 23 roles compared to 18). It includes a broader range of specialized roles such as Research nurse, Home care, Phoniatrician, Acupuncturist (for saliva stimulation), Sexologist/fertility expert and Lymphatic therapist. In contrast, the English list includes an ‘Oral rehabilitation team’ that is not formalized within the Swedish healthcare system.

## 5 Conclusion

In conclusion, by following the systematic methodology described by ISPOR, a translated and culturally adapted version of the PCI‐HN is now available in Swedish. Both patients and healthcare professionals participating in the study highlighted its usefulness, and the PCI‐HN received excellent ratings for content validity. The next step will be to successfully introduce the Swedish PCI‐HN into the healthcare setting to be able to support patients with HNC and their caregivers in raising issues related to the disease and treatment, both during treatment and during the post‐treatment period. Long‐term, the information transfer to patients should be increased and individualized, enabling more streamlined and effective consultations and improved outcomes.

The Swedish translated and culturally adapted PCI‐HN AD and PCI‐HN PT can be obtained free of charge from the corresponding author.

## Author Contributions

Conceptualization (Y.T.E., S.E.); Methodology (Y.T.E., S.E., G.L., L.T., C.F.); Investigation ISPOR Step 7 (Y.T.E.); Formal analysis ISPOR Steps 1 to 6 (Y.T.E., S.E., G.L., L.T., C.F.); Formal analysis ISPOR Step 7 (Y.T.E., S.E.); Formal analysis ISPOR Steps 8 to 9 (Y.T.E., S.E., G.L., L.T., C.F.); Writing – original draft (Y.T.E., S.E.); Writing – review and editing (G.L., S.N.R., L.T., C.F.). All authors approved the final manuscript.

## Funding

This work was supported by The Swedish Cancer Society (grant number 211419 Pj) and The Sjöberg foundation (grant number 2021‐316).

## Ethics Statement

This study was performed in line with the principles of the Declaration of Helsinki. Approval was granted by the Swedish Ethical Review Authority (Dnr 2024–00281‐01).

## Consent

Written informed consent was obtained from all individual participants included in the study.

## Conflicts of Interest

The PCI‐HN instrument was developed by SR. None of the remaining co‐authors have any conflicts of interest to declare.

## Data Availability

The data that support the findings of this study are available on request from the corresponding author. The data are not publicly available due to privacy or ethical restrictions.

## References

[1] A. Argiris , M. V. Karamouzis , D. Raben , and R. L. Ferris , “Head and Neck Cancer,” Lancet 371, no. 9625 (2008): 1695–1709, 10.1016/S0140-6736(08)60728-X. PMC7720415 18486742

[2] Regionala Cancercentrum i Samverkan , “National Care Programme for Head and Neck Cancer,” 2024, https://cancercentrum.se/samverkan/cancerdiagnoser/huvud‐och‐hals/vardprogram/.

[3] A. Vissink , J. Jansma , F. Spijkervet , F. Burlage , and R. Coppes , “Oral Sequelae of Head and Neck Radiotherapy,” Critical Reviews in Oral Biology and Medicine 14, no. 3 (2003): 199–212, 10.1177/154411130301400305. 12799323

[4] A. Trotti , “Toxicity in Head and Neck Cancer: A Review of Trends and Issues,” International Journal of Radiation Oncology, Biology, Physics 47, no. 1 (2000): 1–12, 10.1016/s0360-3016(99)00558-1. 10758302

[5] A. Y. Chen , R. Frankowski , J. Bishop‐Leone , et al., “The Development and Validation of a Dysphagia‐Specific Quality‐Of‐Life Questionnaire for Patients With Head and Neck Cancer: The MD Anderson Dysphagia Inventory,” Archives of Otolaryngology – Head & Neck Surgery 127, no. 7 (2001): 870–876. 11448365

[6] M. E. Lokker , M. P. Offerman , L. A. van der Velden , M. F. de Boer , J. F. Pruyn , and S. C. Teunissen , “Symptoms of Patients With Incurable Head and Neck Cancer: Prevalence and Impact on Daily Functioning,” Head & Neck 35, no. 6 (2013): 868–876, 10.1002/hed.23053. 22714991

[7] J. Ringash , L. J. Bernstein , D. Cella , et al., “Outcomes Toolbox for Head and Neck Cancer Research,” Head & Neck 37, no. 3 (2015): 425–439, 10.1002/hed.23561. 24318166

[8] C. Hammermüller , A. Hinz , A. Dietz , et al., “Depression, Anxiety, Fatigue, and Quality of Life in a Large Sample of Patients Suffering From Head and Neck Cancer in Comparison With the General Population,” BMC Cancer 21 (2021): 1–11, 10.1186/s12885-020-07773-6. PMC7825198 33482771

[9] Y. T. Ehrsson , P. Fransson , and S. Einarsson , “Mapping Health‐Related Quality of Life, Anxiety, and Depression in Patients With Head and Neck Cancer Diagnosed With Malnutrition Defined by GLIM,” Nutrients 13, no. 4 (2021): 1167, 10.3390/nu13041167. PMC8066581 33916049

[10] E. A. Theunissen , S. C. Bosma , C. L. Zuur , et al., “Sensorineural Hearing Loss in Patients With Head and Neck Cancer After Chemoradiotherapy and Radiotherapy: A Systematic Review of the Literature,” Head & Neck 37, no. 2 (2015): 281–292, 10.1002/hed.23551. 24478269

[11] C. Sioka and A. P. Kyritsis , “Central and Peripheral Nervous System Toxicity of Common Chemotherapeutic Agents,” Cancer Chemotherapy and Pharmacology 63 (2009): 761–767, 10.1007/s00280-008-0876-6. 19034447

[12] S. Einarsson , G. Laurell , and Y. Tiblom Ehrsson , “Experiences and Coping Strategies Related to Food and Eating up to Two Years After the Termination of Treatment in Patients With Head and Neck Cancer,” European Journal of Cancer Care (English Edition) 28, no. 2 (2019): e12964, 10.1111/ecc.12964. 30444049

[13] S. Rogers , J. El‐Sheikha , and D. Lowe , “The Development of a Patients Concerns Inventory (PCI) to Help Reveal Patients Concerns in the Head and Neck Clinic,” Oral Oncology 45, no. 7 (2009): 555–561, 10.1016/j.oraloncology.2008.09.004. 19028135

[14] A. Kanatas , D. Lowe , and S. N. Rogers , “The Patient Concerns Inventory in Head and Neck Oncology: A Structured Review of Its Development, Validation and Clinical Implications,” European Archives of Oto‐Rhino‐Laryngology 279, no. 11 (2022): 5097–5111, 10.1007/s00405-022-07499-0. PMC9519723 35842858

[15] J. Hatta , J. Doss , and S. Rogers , “The Feasibility of Using Patients Concerns Inventory (PCI) in Managing Malaysian Oral Cancer Patients,” International Journal of Oral and Maxillofacial Surgery 43, no. 2 (2014): 147–155, 10.1016/j.ijom.2013.08.006. 24074487

[16] C. Shunmugasundaram , C. Rutherford , P. N. Butow , P. Sundaresan , and H. M. Dhillon , “Content Comparison of Unmet Needs Self‐Report Measures Used in Patients With Head and Neck Cancer: A Systematic Review,” Psycho‐Oncology 28, no. 12 (2019): 2295–2306, 10.1002/pon.5257. 31654540

[17] S. N. Rogers , C. Allmark , F. Bekiroglu , et al., “Improving Quality of Life Through the Routine Use of the Patient Concerns Inventory for Head and Neck Cancer Patients: Main Results of a Cluster Preference Randomised Controlled Trial,” European Archives of Oto‐Rhino‐Laryngology 278 (2021): 3435–3449, 10.1007/s00405-020-06533-3. PMC7751263 33346856

[18] S. N. Rogers , A. Alvear , A. Anesi , et al., “Variations in Concerns Reported on the Patient Concerns Inventory in Patients With Head and Neck Cancer From Different Health Settings Across the World,” Head & Neck 42, no. 3 (2020): 498–512, 10.1002/hed.26027. PMC7079080 31833121

[19] D. Wild , A. Grove , M. Martin , et al., “Principles of Good Practice for the Translation and Cultural Adaptation Process for Patient‐Reported Outcomes (PRO) Measures: Report of the ISPOR Task Force for Translation and Cultural Adaptation,” Value in Health 8, no. 2 (2005): 94–104, 10.1111/j.1524-4733.2005.04054.x. 15804318

[20] R. A. Krueger and M. A. Casey , Focus Groups: A Practical Guide for Applied Research, Fifth ed. (Sage Publications, 2014).

[21] D. F. Polit and C. T. Beck , “The Content Validity Index: Are You Sure You Know What's Being Reported? Critique and Recommendations,” Research in Nursing & Health 29, no. 5 (2006): 489–497, 10.1002/nur.20147. 16977646

[22] D. F. Polit , C. T. Beck , and S. V. Owen , “Is the CVI an Acceptable Indicator of Content Validity? Appraisal and Recommendations,” Research in Nursing & Health 30, no. 4 (2007): 459–467, 10.1002/nur.20199. 17654487

[23] R. Elaldi , L.‐M. Roussel , J. Gal , et al., “Correlations Between Long‐Term Quality of Life and Patient Needs and Concerns Following Head and Neck Cancer Treatment and the Impact of Psychological Distress. A Multicentric Cross‐Sectional Study,” European Archives of Oto‐Rhino‐Laryngology 278 (2021): 2437–2445, 10.1007/s00405-020-06326-8. 32901366

[24] C. Kubrak , K. Olson , and V. E. Baracos , “The Head and Neck Symptom Checklist: An Instrument to Evaluate Nutrition Impact Symptoms Effect on Energy Intake and Weight Loss,” Supportive Care in Cancer 21 (2013): 3127–3136, 10.1007/s00520-013-1870-z. 23852426

[25] Y. Tiblom Ehrsson , S. Einarsson , P. Fransson , and G. Laurell , “Swedish Translation and Cultural Adaptation of the Head and Neck Patient Symptom Checklist: An Instrument to Screen for Nutrition Impact Symptoms in Clinical Practice and Research,” Western Journal of Nursing Research 46 (2024): 790–798, 10.1177/01939459241274342. PMC11457587 39180372

[26] J. Johnson , S. Carlsson , M. Johansson , et al., “Development and Validation of the Gothenburg Trismus Questionnaire (GTQ),” Oral Oncology 48, no. 8 (2012): 730–736, 10.1016/j.oraloncology.2012.02.013. PMC5479349 22418239

[27] M. Johansson , T. Karlsson , and C. Finizia , “Further Validation of the Gothenburg Trismus Questionnaire (GTQ),” PLoS One 15, no. 12 (2020): e0243805, 10.1371/journal.pone.0243805. PMC7746186 33332402

[28] S. Carlsson , A. Rydén , I. Rudberg , M. Bove , H. Bergquist , and C. Finizia , “Validation of the Swedish MD Anderson Dysphagia Inventory (MDADI) in Patients With Head and Neck Cancer and Neurologic Swallowing Disturbances,” Dysphagia 27 (2012): 361–369, 10.1007/s00455-011-9375-8. 22105944

[29] L. Tuomi , P. Fransson , J. Wennerberg , and C. Finizia , “A Longitudinal Study of the Swedish MD Anderson Dysphagia Inventory in Patients With Oral Cancer,” Laryngoscope Investigative Otolaryngology 5, no. 6 (2020): 1125–1132, 10.1002/lio2.490. PMC7752062 33364403

[30] N. K. Aaronson , S. Ahmedzai , B. Bergman , et al., “The European Organization for Research and Treatment of Cancer QLQ‐C30: A Quality‐Of‐Life Instrument for Use in International Clinical Trials in Oncology,” Journal of the National Cancer Institute 85, no. 5 (1993): 365–376, 10.1093/jnci/85.5.365. 8433390

[31] A. Mortensen , I. Wessel , S. N. Rogers , A. Tolver , and M. Jarden , “Needs Assessment in Patients Surgically Treated for Head and Neck Cancer—A Randomized Controlled Trial,” Supportive Care in Cancer 30, no. 5 (2022): 4201–4218, 10.1007/s00520-021-06759-9. 35083545

[32] S. Rogers , F. Thomson , and D. Lowe , “The Patient Concerns Inventory Integrated as Part of Routine Head and Neck Cancer Follow‐Up Consultations: Frequency, Case‐Mix, and Items Initiated by the Patient,” Annals of the Royal College of Surgeons of England 100, no. 3 (2018): 209–215, 10.1308/rcsann.2017.0215. PMC5930095 29364010

[33] V. S. Ezeofor , L. H. Spencer , S. N. Rogers , et al., “An Economic Evaluation Supported by Qualitative Data About the Patient Concerns Inventory (PCI) Versus Standard Treatment Pathway in the Management of Patients With Head and Neck Cancer,” PharmacoEconomics ‐ Open 6, no. 3 (2022): 389–403, 10.1007/s41669-021-00320-4. PMC8802252 35099783

[34] S. N. Rogers , C. Y. Chieng , A. Davies , A. Aziz , and D. Lowe , “Health‐Related Quality of Life and Concerns in Patients Attending an Oral and Maxillofacial Oncology Review Clinic: Late Effects 5‐and 10‐Year Following Cancer Diagnosis,” Frontiers in Oral and Maxillofacial Medicine 4 (2022): 22.

[35] N. Ghazali , B. Roe , D. Lowe , et al., “Using the Patients Concerns Inventory for Distress Screening in Post‐Treatment Head and Neck Cancer Survivors,” Journal of Cranio‐Maxillo‐Facial Surgery 45, no. 10 (2017): 1743–1748, 10.1016/j.jcms.2017.07.009. 28844398

[36] S. Rogers , D. Lowe , and A. Kanatas , “Suitability of the Patient Concerns Inventory as a Holistic Screening Tool in Routine Head and Neck Cancer Follow‐Up Clinics,” British Journal of Oral & Maxillofacial Surgery 54, no. 4 (2016): 415–421, 10.1016/j.bjoms.2016.01.018. 26919767

[37] S. N. Rogers , C. Semple , G. M. Humphris , D. Lowe , and A. Kanatas , “Using a Patient Prompt List to Raise Concerns in Oncology Clinics Does Not Necessarily Lead to Longer Consultations,” British Journal of Oral & Maxillofacial Surgery 58, no. 9 (2020): 1164–1171, 10.1016/j.bjoms.2020.08.035. 32921505

[38] M. Jefford and M. H. Tattersall , “Informing and Involving Cancer Patients in Their Own Care,” Lancet Oncology 3, no. 10 (2002): 629–637, 10.1016/s1470-2045(02)00877-x. 12372725

[39] V. Jenkins , L. Fallowfield , and J. Saul , “Information Needs of Patients With Cancer: Results From a Large Study in UK Cancer Centres,” British Journal of Cancer 84, no. 1 (2001): 48–51, 10.1054/bjoc.2000.1573. PMC2363610 11139312

[40] A. Ferri , G. Lilloni , G. Molteni , et al., “The Psychosocial Needs of Head and Neck Cancer Patients: A Multicenter Study,” European Archives of Oto‐Rhino‐Laryngology 281 (2024): 4913–4920, 10.1007/s00405-024-08680-3. 38704510

[41] M. E. Mills and K. Sullivan , “The Importance of Information Giving for Patients Newly Diagnosed With Cancer: A Review of the Literature,” Journal of Clinical Nursing 8, no. 6 (1999): 631–642, 10.1046/j.1365-2702.1999.00296.x. 10827609

[42] C. Charles , A. Gafni , and T. Whelan , “Shared Decision‐Making in the Medical Encounter: What Does It Mean? (Or It Takes at Least Two to Tango),” Social Science & Medicine 44, no. 5 (1997): 681–692, 10.1016/s0277-9536(96)00221-3. 9032835

[43] M. Gattellari , P. N. Butow , and M. H. Tattersall , “Sharing Decisions in Cancer Care,” Social Science & Medicine 52, no. 12 (2001): 1865–1878, 10.1016/s0277-9536(00)00303-8. 11352412

[44] R. L. Street, Jr. , “Information‐Giving in Medical Consultations: The Influence of Patients' Communicative Styles and Personal Characteristics,” Social Science & Medicine 32, no. 5 (1991): 541–548, 10.1016/0277-9536(91)90288-n. 2017721

